# Prospective Acceptability and Implementation Considerations of a Proposed Brief Online Mindfulness-Based Cognitive Therapy Intervention Among Military Personnel

**DOI:** 10.3390/bs16101821

**Published:** 2026-10-05

**Authors:** Marcelo Alexandre Cabaça Matias, Pedro Joel Rosa

**Affiliations:** 1 HEI-Lab: Digital Human-Environment Interaction Labs, Lusófona University, Campo Grande, 376, 1749-024 Lisboa, Portugal; 2 Experimental Psychology Lab, Instituto Superior Manuel Teixeira Gomes (ISMAT), 8500-590 Portimão, Portugal

**Keywords:** mindfulness-based cognitive therapy, burnout, military personnel, acceptability

## Abstract

Burnout constitutes a critical psychological and operational challenge in military environments, with potential implications for well-being and occupational functioning. Mindfulness-Based Cognitive Therapy has shown beneficial effects on stress and burnout across different populations; however, little is known about how brief online adaptations are perceived by active-duty military personnel prior to implementation. This qualitative study explored the prospective acceptability of a proposed brief online Mindfulness-Based Cognitive Therapy program and identified perceived implementation considerations within the Portuguese Army. Fifteen participants (11 men and 4 women) took part in semi-structured interviews, which were analyzed using reflexive thematic analysis within a constructivist framework. Five themes were identified: previous experiences with psychological services, prospective acceptability of the proposed program, practical and cultural barriers, conditions for implementation, and preferences regarding format and facilitation. Participants generally viewed the proposed program favorably and identified brevity, confidentiality, scheduling flexibility, digital access, leadership support, and facilitator credibility as potentially important for future implementation, while stigma and workload were perceived as relevant barriers. These findings reflect prospective perceptions rather than actual intervention feasibility or effectiveness. *Public Significance Statement:* This study identifies key factors influencing how active-duty military personnel perceive a proposed brief online mindfulness-based intervention before implementation.

## 1. Introduction

Mindfulness-based interventions (MBIs) are structured therapeutic programs that integrate mindfulness meditation practices into clinical and organizational contexts, aiming to improve psychological well-being and alleviate stress-related symptomatology (Lomas et al., 2019). Accumulating evidence indicates that MBIs can reduce symptoms of depression, anxiety, stress, and burnout, supporting their use across both clinical and occupational contexts (Pérez et al., 2022).

Among the various MBIs, Mindfulness-Based Cognitive Therapy (MBCT) has emerged as one of the most widely applied and well-validated protocols. Originally developed in the late 1990s to prevent relapses in recurrent depression (Sears, 2015), MBCT combines mindfulness training with cognitive-behavioral strategies to cultivate present-moment awareness and disrupt maladaptive cycles of rumination. Its application has subsequently extended beyond recurrent depression, with evidence suggesting potential benefits for stress, burnout, emotional regulation, and psychological functioning (Wang et al., 2023; Chan et al., 2021).

However, the standard structure of mindfulness-based programs may limit their suitability for occupational groups characterized by high workloads and limited scheduling flexibility. Traditional MBIs are commonly delivered over approximately eight weeks, with lengthy weekly sessions and substantial home practice requirements (Parsons et al., 2017). These demands have encouraged the development of shorter and digitally delivered formats intended to preserve core mindfulness processes while reducing the burden associated with participation (Birtwell et al., 2019).

Brief MBIs typically comprise four to seven sessions and seek to retain therapeutic processes such as attentional regulation, decentering, and acceptance while reducing overall time demands (Birtwell et al., 2019). Evidence from occupational settings suggests that abbreviated programs may offer a practical alternative when organizational and scheduling constraints make standard formats difficult to implement. For example, Luberto et al. (2017) reported reductions in stress and burnout following a four-week MBCT program among hospital staff, while Demarzo et al. (2017) found that a four-week MBI produced outcomes comparable to a standard eight-week protocol. Similarly, digitally delivered mindfulness programs may increase accessibility where flexibility and continuity of participation are important considerations (Witarto et al., 2022).

These considerations are particularly relevant in military settings, where personnel operate within highly structured organizations and may experience substantial occupational and operational demands. Military personnel may be exposed to sustained workload, irregular schedules, operational pressures, and psychologically demanding experiences, making the accessibility and contextual fit of psychological interventions especially important. Mental health difficulties documented in military populations include burnout and posttraumatic stress disorder, among other conditions (Hosseini et al., 2023; Watkins et al., 2018).

Regarding burnout, a systematic review by Hosseini et al. (2023) estimated that EE prevalence ranged up to 49.7%, DP up to 59.6%, and low personal accomplishment up to 60% in military samples. Within the Portuguese Army, Matias and Rosa (2025) likewise identified clinically relevant levels of burnout dimensions among personnel, reinforcing the importance of preventive approaches that are compatible with military occupational demands. Burnout in military settings has also been associated with outcomes relevant to occupational functioning, including reduced unit cohesion, impaired performance, and increased risk-taking behaviors (Shi et al., 2024).

Mindfulness-based approaches have increasingly been examined in military and veteran populations. Evidence suggests potential benefits for stress regulation, attentional control, emotional regulation, and trauma-related distress (Goldberg et al., 2020; Schuman et al., 2023). Digital delivery may be particularly relevant in this context because it can reduce geographical and scheduling constraints. For example, Best et al. (2020) reported reductions in burnout and stress following a mobile-delivered mindfulness intervention among military nurse practitioners. Research with veterans has also indicated generally favorable perceptions of mindfulness practices and digitally delivered mindfulness resources (Colgan et al., 2017; Reyes et al., 2020; Wielgosz et al., 2025).

The Portuguese Army represents a particularly relevant context for examining the prospective implementation of brief psychological interventions. Military service is characterized by hierarchical organizational structures, variable operational demands, irregular schedules, and expectations of continuous availability, all of which may influence participation in psychological programs. In addition, concerns related to stigma, confidentiality, and help seeking may affect how mental health interventions are perceived within military settings (Sharp et al., 2015; Fikretoglu et al., 2022). These contextual characteristics reinforce the importance of examining whether a brief and digitally delivered mindfulness-based intervention is perceived as acceptable and practically compatible with the realities of active-duty service before formal implementation.

Nevertheless, much of the existing literature has focused on intervention outcomes or on veteran populations rather than on how active-duty personnel perceive the prospective use of brief online mindfulness interventions within their organizational context. Evidence of therapeutic benefit alone does not establish whether an intervention will be viewed as acceptable or compatible with the practical and cultural characteristics of a military organization. Understanding these perceptions before implementation is therefore particularly relevant when interventions must accommodate occupational demands, organizational structures, and the specific context in which personnel would be expected to participate.

This gap is especially relevant within the Portuguese Army, where evidence concerning burnout has begun to emerge (Matias & Rosa, 2025, 2026), but little is known about how active-duty personnel perceive brief online mindfulness-based interventions or the conditions that they consider important for their implementation. Examining these perspectives may therefore help determine whether such an intervention is perceived as relevant and contextually appropriate before proceeding to formal intervention testing.

In the present study, acceptability is considered prospectively, referring to participants anticipated cognitive and emotional responses to the proposed intervention before direct exposure to it (Sekhon et al., 2017). Perceived feasibility refers to participants views regarding whether the proposed program could be practically delivered within the organizational and operational conditions of the Portuguese Army, whereas implementation considerations encompass the perceived barriers, facilitators, and contextual conditions that may influence future delivery (Proctor et al., 2011). Because participants did not directly use or receive the intervention, usability and actual feasibility were not evaluated.

Therefore, the present study aims to explore the prospective acceptability of a proposed brief online MBCT program among Portuguese Army personnel and to identify perceived feasibility and implementation considerations relevant to its future delivery in an active-duty military setting.

## 2. Method

This study adopted a qualitative research design grounded in a constructivist paradigm, consistent with counseling research that seeks to understand how individuals construct meaning within their professional and organizational contexts. An interpretive qualitative approach was employed using individual semi-structured in-depth interviews to explore participants perceptions of, and meanings attributed to, a proposed brief online MBCT program within the Portuguese Army. This approach assumes that participants views are shaped by their personal experiences in the military, as well as by the organizational and cultural context in which they operate. Accordingly, the aim of the research was to understand how participants interpreted and attributed meaning to the proposed program and its potential implementation within the Portuguese Army. This perspective guided all stages of the study, including data collection, analysis, and the presentation of findings. This study is reported in accordance with the *Journal Article Reporting Standards for Qualitative Research* (JARS–Qual) of the American Psychological Association (American Psychological Association, 2020; see Supplemental Item S1).

### 2.1. Ethics

Ethical approval for the study was obtained from the Ethics and Deontology Committee for Scientific Research (CEDIC) of Lusófona University prior to data collection, under the grant CEDIC-2025-6-34. Authorization was also granted by the Chief of Staff of the Portuguese Army through an official written statement. All participants were provided with detailed information about the purpose and procedures of the study before participation. Written informed consent was obtained from each participant. Participation was voluntary, with the option to withdraw at any time without penalty. Participants could also choose to omit responses to any questions they prefer not to answer. Confidentiality was protected through pseudonymization, with participants identified in transcripts and subsequent analyses using numerical codes (e.g., ID1, ID2) rather than names. Interview audio was recorded using the principal investigators personal mobile device and was not transferred to a computer or stored through Microsoft Teams. Recordings were accessed only for transcription purposes and were permanently deleted from the recording device once transcription had been completed. Pseudonymized transcripts and research files were stored in password-protected files on the principal investigator’s personal computer, with access restricted to the research team. Data handling procedures were conducted in accordance with the General Data Protection Regulation.

### 2.2. Recruitment and Data Collection

Participants were recruited using a non-probabilistic convenience sampling strategy. Recruitment was conducted through three on-site visits to military units, during which potential participants were informed about the study, as well as through institutional contact with service members who had previously expressed interest in participating in future research. A total of 15 individuals were approached and all agreed to participate, resulting in no refusals. Five participants were included during each recruitment visit, although some interviews were subsequently conducted online according to participant availability. Three participants had previously participated in earlier studies conducted by the research team. Participants were not recruited based on burnout symptoms or burnout status, and burnout was not assessed as part of the present study. The proposed intervention was introduced as a program potentially relevant to stress and burnout in military personnel, and participants were invited to comment on its perceived relevance and potential usefulness within that context.

Those who expressed interest received detailed study information, including the purpose, procedures, and confidentiality assurances, and were asked to provide written informed consent prior to participation, either in person or electronically for those completing online interviews. No monetary incentives or compensation were provided for participation. The sample size was considered appropriate for an in-depth qualitative exploration focused on participants perceptions within a specific organizational and cultural context. Recruitment and preliminary engagement with the data occurred concurrently, allowing the research team to assess the depth, diversity, and relevance of the accounts generated in relation to the study objectives.

Sociodemographic and organizational characteristics of participants are presented in Table 1.

### 2.3. Interview Procedures

Individual semi-structured interviews were conducted by the principal investigator, a male PhD candidate with formal training in qualitative research methods and previous academic experience investigating burnout within military populations. The interviewer was also an active-duty military officer; however, no prior personal relationships existed between the researcher and any of the participants, and the researcher held no supervisory authority over them. Participants were explicitly informed that participation, non-participation, and the responses provided during the interview would have no influence on their military career, performance evaluations, or professional standing. The researchers dual role as both a military officer and researcher was acknowledged as a potentially relevant influence on the interview process, particularly when discussing sensitive issues related to psychological support, leadership, stigma, and confidentiality. Reflexivity was therefore maintained throughout data collection through contextual and reflexive notes, documentation of analytic decisions, and regular supervision focused on critically examining how the researchers’ institutional position could shape interactions with participants and subsequent interpretation of the data.

The brief online MBCT program used as the reference framework for this study, upon which the interview guide was developed (see Supplemental Item S2), was structured according to the four-week adaptation proposed by Luberto et al. (2017; see Supplemental Item S3) and served to anchor participants reflections during the interviews rather than to function as an intervention being tested for efficacy. This adaptation was derived from the original MBCT protocol developed by Segal et al. (2002) and later adapted for stress-related conditions by Sears (2015). Luberto et al. (2017) reported improvements in self-regulation and mindfulness competencies, alongside reductions in stress and burnout among healthcare professionals. Similar findings have been reported by OBryan et al. (2023), who observed reductions in anxiety and transdiagnostic processes using the same protocol. Participants did not receive, participate in, or complete the proposed MBCT program. The program description was presented solely as a reference framework to support discussion during the interviews. Accordingly, participant responses reflected prospective perceptions of the proposed intervention rather than experiences arising from direct exposure to it.

The proposed intervention was conceptualized as a brief adapted MBCT program rather than a full delivery of the standard MBCT protocol. It retained core MBCT elements, including mindfulness practice, present moment awareness, guided inquiry, reflection on habitual cognitive and emotional responses, and the application of mindfulness skills to daily experiences. However, the intervention used was substantially abbreviated, particularly in relation to overall program duration, session length, and home practice requirements. The use of a shorter format was informed by previous research indicating that abbreviated mindfulness-based programs can produce meaningful psychological benefits while reducing the time demands associated with standard interventions (Luberto et al., 2017; Demarzo et al., 2017). The program comprised one synchronous online session per week, each lasting approximately one hour, over a four-week period. Each session included two to three mindfulness exercises, guided inquiry, reflection on the application of mindfulness strategies to daily experiences, and a brief review component. Home practice was intentionally limited to a maximum of 30 min per week to reduce participation burden and increase compatibility with demanding and variable occupational schedules.

The interview guide included open ended questions addressing predefined domains relevant to the study objectives, including perceived acceptability, implementation barriers and facilitators, confidentiality, leadership support, scheduling flexibility, delivery format, facilitator characteristics, and prospective relevance to stress and burnout. These domains provided a structured framework for the interviews while allowing participants to elaborate freely, introduce additional concerns, and develop perspectives beyond the predefined topics. Because stress and burnout were explicitly included in the study framing and interview guide, participants responses concerning potential benefits in these areas should be understood as prompted expectations rather than independent evidence of intervention appropriateness or effectiveness.

Interviews were conducted in September 2025, either in person at military units or online via Microsoft Teams for participants unable to attend in person. In person interviews were conducted in a closed and private room, with only the participant and the researcher present. For online interviews, participants were instructed to join from a private location where no other individuals had access to the conversation, and the researcher likewise conducted the interview from a private setting. The researcher used a personal Microsoft Teams account, while participants accessed the interview directly through a link without the need to create or use a Microsoft Teams account. No recording function within Microsoft Teams was used. Audio was recorded separately using the principal investigators personal mobile device, with participants consent. Each interview lasted approximately 30 to 45 min, and no observers or nonparticipants were present.

### 2.4. Data Analysis

Audio recordings were transcribed verbatim and analyzed using reflexive thematic analysis, consistent with the constructivist and interpretive orientation of the study. Analysis followed the six phases described by Braun and Clarke (2006, 2021): familiarization with the data, generation of initial codes, development of candidate themes, review and refinement of themes, definition and naming of themes, and production of the analytic narrative. Transcripts were read and re read to support immersion in the data and sensitivity to participants meanings and contextual nuances. Theme development was iterative and interpretive, with codes and candidate themes revisited throughout the analytic process as the researcher engaged more deeply with the dataset. Interviews were conducted and transcribed in Portuguese, and coding and thematic analysis were also performed in Portuguese to preserve participants original meanings and contextual nuances. Only the quotations selected for inclusion in the manuscript were translated into English after the analytic process. The principal investigator translated the selected excerpts, and the translations were reviewed by the study supervisor against the original Portuguese quotations to ensure semantic accuracy and consistency with participants intended meanings.

Data management and coding were performed using ATLAS.ti (version 23, Lumivero, Denver, CO, USA). Coding combined a deductive component, informed by the predefined interview domains and research questions, with an inductive component that captured meanings, patterns, and issues developed from participants accounts. Accordingly, the analysis did not treat the final thematic structure as entirely emergent from the data. Rather, predefined areas of inquiry provided an initial analytic framework, while the interpretation and refinement of themes and subthemes were developed iteratively through engagement with participants narratives. To support analytic rigor and reflexivity, the coding process and thematic structure were discussed collaboratively with the study supervisor through regular peer debriefing sessions, with particular attention to alternative interpretations and the potential influence of the principal investigators’ positionality on the analytic process.

An audit trail was maintained to document coding decisions, theme refinement, and reflexive notes throughout the analytic process. Methodological rigor was supported through verbatim transcription, systematic documentation of analytic decisions, reflexive note taking, and the use of direct participant quotations to ground interpretations in the data. These procedures were used to enhance transparency, analytic coherence, and reflexivity throughout the research process. The interpretation of findings was also considered in relation to the study objectives and existing literature on mindfulness-based interventions in military contexts. Representative excerpts for each theme are presented in Section 3.

Transcripts were not returned to participants because participant validation was not incorporated into the reflexive thematic analysis design and to avoid additional circulation of potentially identifiable qualitative material within a sensitive institutional context.

## 3. Results

Fifteen active-duty Portuguese Army personnel (11 men, 4 women; M = 40 years, SD = 12.9) participated in individual interviews conducted in September 2025. Participants mean service time was 18.4 years (SD = 12.8) and ranks were evenly distributed among enlisted, non-commissioned officers, and officers.

Reflexive thematic analysis identified five major themes describing participants prospective perceptions of the proposed brief online MBCT program and the contextual conditions relevant to its potential implementation, as shown in Table 2. Representative participant quotations are presented throughout Section 3 to illustrate the interpretation of each theme. As participants had not received or experienced the proposed intervention, the findings presented below reflect their prospective perceptions and expectations regarding the program and its potential implementation.

### 3.1. Experience and Contact with Psychological Services

Participants described heterogeneous experiences regarding contact with psychological support. For many, psychological care was not part of their military experience or culture, several admitted they had never sought psychological assistance: *“Ive never had psychological follow-up, even though I have felt the need a few times”* (ID1, age 30 years). Others mentioned that they knew colleagues who had done so and found it helpful: *“I never went myself, but I know people who did, and they said it helped them a lot”* (ID5, age 40 years).

Some participants reported indirect contact through training or specific missions involving psychologists, this exposure seemed to reduce hesitation: *“I had contact with psychology during a training course, and it made me realize how much these areas matter for our job”* (ID15, age 21 years).

### 3.2. Prospective Acceptability of the Proposed Brief Online MBCT

Across interviews, participants generally described the proposed brief online MBCT program as potentially appropriate, practical, and relevant to the military context. Its short duration, structured format, and digital accessibility were identified as features that could facilitate its future implementation among active-duty personnel.

Participants repeatedly emphasized that the four-week structure was realistic and compatible with the rhythm of military service, the concise and goal-oriented design was regarded as particularly appealing in contrast to longer interventions requiring extensive commitment. As one participant explained, *“Four sessions are perfect, short, straightforward, and easy to follow”* (ID8, age 55 years). This perceived balance between program length and prospectivebenefits contributed to participants views that the proposed format could be compatible with heavy workloads and unpredictable schedules.

The practical orientation of the program, emphasizing direct application to stress management, was also a major source of acceptability, participants saw the content as connected to real operational challenges rather than abstract psychological concepts. One participant noted, *“how to use breathing before a shooting exercise, or helping to deal with accumulated tension after long periods of service, whether physical and/or psychological”* (ID10, age 49 years). Others described it as a valuable tool for developing self-awareness and emotional regulation in demanding work environments, suggesting that mindfulness training could potentially support composure, focus, and adaptability under pressure: *“less mistakes, less accidents, greater job satisfaction, and increased capabilities”* (ID3, age 50 years).

The online modality was considered one of the programs most attractive features, minimizing logistical obstacles such as travel or schedule coordination: *“The flexibility is a big advantage”* (ID9, age 52 years). For many participants, the synchronous online format and the possibility of attending sessions remotely were viewed as potentially facilitating compatibility with irregular duty hours, deployments, and rotating shifts. Some participants also suggested that greater scheduling flexibility or alternative access arrangements could further support participation when attendance at a scheduled synchronous session was not possible. Participants also perceived this flexibility as potentially supporting participation and providing greater personal control over engagement with the program, which some considered particularly relevant within a hierarchical institutional context.

Another recurring theme concerned confidentiality and psychological safety, which were identified as crucial for acceptability. Participants believed that the program private and individual nature would reduce reluctance to participate, as stated: *“What is important is that its private, if others know you are doing it, many will not take part”* (ID10, age 49 years). This sentiment was echoed across several interviews, reinforcing that privacy, confidentiality, and discretion were perceived as important conditions for participation and open engagement.

In addition, participants highlighted the simplicity and clarity of the proposed program as factors contributing to its perceived contextual fit. *“It is a practical and dynamic program. You can understand its purpose right from the start”* (ID7, age 51 years). This accessibility was perceived as potentially facilitating the integration of mindfulness practices into daily routines.

Several participants also recognized that the program aligned well with military values such as discipline, self-regulation, and operational readiness. Several participants framed mindfulness less as a form of therapy and more as a potential training tool for enhancing cognitive and emotional performance. In this sense, mindfulness practice was reframed as a method to strengthen concentration and resilience, resonating with the Army’s focus on efficiency and endurance. One participant explained that such programs could: *“help soldiers recover mental balance after intense missions and improve overall operational focus”* (ID9, age 52 years).

Collectively, these perspectives indicate generally favorable prospectiveperceptions of the proposed program and suggest that its brevity, structure, online accessibility, and emphasis on self-regulation were viewed as potentially compatible with military routines and values. Participants also described the proposed intervention in terms of potential personal and professional benefits; however, these accounts represent expectations rather than demonstrated intervention outcomes.

### 3.3. Practical and Cultural Barriers

Although participants generally recognized the potential benefits of the brief online MBCT program, they described multiple cultural and institutional barriers that could undermine participation. A recurring issue was the stigma associated with seeking psychological support within the military, several participants noted that mental health remains a sensitive topic rarely discussed among peers. As one participant explained, *“Some people here still see psychological help as something for those who are weak or unstable”* (ID13, age 25 years). Another participant elaborated that this perception discourages individuals from engaging in such programs: *“If others know you are doing something related to psychology, they start making comments, it is easier to just avoid it”* (ID15, age 21 years).

Participants described how prospectivejudgment and mistrust could discourage personnel from openly expressing interest in mental health interventions, fearing repercussions or being perceived as less competent. One participant noted, *“Theres always that idea that if you need psychological help, you are not fit for service”* (ID11, age 22 years). Others highlighted that stigma persists not only among soldiers but also within leadership structures, which often fail to promote open conversations about emotional well-being: *“It is not usual for the people to talk about it”* (ID14, age 31 years).

Alongside stigma, participants described structural barriers such as demanding work schedules, limited time, and lack of flexibility. *“Our timetable changes sometimes every day. It is hard to guarantee that you will have that moment to stop”* (ID7, age 51 years). Others mentioned that the operational culture prioritizes availability and resilience over self-care, reinforcing reluctance to engage: *“Were expected to be always ready. There is no much room for the I”* (ID6, age 60 years).

Privacy also emerged as a key concern. Participants expressed that, within military environments, personal activities are often subject to observation and comment: *“Theres no privacy in the unit. If you are doing something different, everyone notices and asks why are you doing it”* (ID13, age 25 years).

Collectively, these narratives identify stigma, demanding schedules, privacy concerns, and perceived lack of leadership support as potential barriers to future implementation within the Portuguese Army.

### 3.4. Conditions for Implementation

Participants identified formal authorization, leadership endorsement, and organizational flexibility as potentially important conditions for future implementation. Institutional support was repeatedly described as the foundation for credibility: *“If there is an established protocol and it receives command approval, participation will follow.”* (ID1, age 30 years).

Scheduling flexibility was identified as potentially important for facilitating participation: *“If a soldier is on duty or in an exercise, being able to reschedule the session would solve everything”* (ID11, age 22 years). Participants believed that official endorsement would transform the program from an optional activity into a legitimate component of professional development: *“If the commander understands the advantages of this and how is it structured (…) it will be a green light for it to be used in the units”* (ID2, age 44 years).

Participants emphasized that leadership endorsement could provide institutional legitimacy while participation should remain voluntary to preserve individual autonomy and willingness to engage: *“It should be voluntary so that those who join really want to do it, not because they are ordered to”* (ID12, age 27 years).

### 3.5. Format and Facilitation Preferences

Most of the participants expressed a preference for an individual and online format, particularly because of perceived convenience and privacy: “*Individual and online would be ideal. You can do it in your own space and do not waste time traveling*” (ID1, age 30 years). However, other participants preferred face to face delivery, emphasizing the value of direct interpersonal contact and engagement.

The facilitators profile was also viewed as an important consideration, since many participants expressed a preference for a military psychologist because of familiarity with military culture and occupational demands, whereas others considered an external facilitator acceptable, particularly when confidentiality and professional independence were emphasized: *“It makes sense if its someone from the Army, who understands our routines and the kind of stress we face”* (ID7, age 51 years). Others mentioned that external facilitators could be acceptable if confidentiality were assured, but the shared experience of military life was generally valued as an advantage: *“A military psychologist would be the best option because they convey identification and validation”* (ID9, age 52 years).

Some participants mentioned that they preferred face-to-face learning because of a commitment issue: *“Being there in person is important for conveying this energy of winning people over to the cause, for conveying that energy of creating the initial enthusiasm.”* (ID4, age 43 years).

## 4. Discussion

The present study explored how active-duty Portuguese Army personnel prospective the acceptability of a proposed brief online MBCT program and identified perceived conditions that may influence its future implementation. Participants identified performance and wellness framing, flexibility in delivery, confidentiality, and facilitator credibility as factors that they perceived as potentially facilitating future participation and engagement with the proposed program. These views represent prospective implementation considerations rather than demonstrated effects on adherence or sustainability. These findings should therefore be interpreted as prospective perceptions rather than evidence of actual intervention acceptability, feasibility, adherence, or effectiveness. Thus, the present study directly addresses participants prospectiveperceptions of the proposed program and identifies considerations for its potential future implementation.

Overall, participants perceived the proposed program as compatible with several characteristics of military service, particularly when presented as brief, structured, and adaptable to occupational demands. This perceived contextual fit is consistent with research in which mindfulness-based interventions have been evaluated in military and other high-demand settings. Yan et al. (2024), for example, reported improvements in resilience, posttraumatic stress symptoms, and posttraumatic growth following a mindfulness-based stress reduction program among military medical students, while Zanesco et al. (2019) found improvements in sustained attention and working memory following mindfulness training in elite service members. Although the present study did not evaluate intervention outcomes, these findings provide relevant context for understanding why participants may have viewed a brief mindfulness-based program as compatible with cognitively and operationally demanding environments.

Brevity and structure appeared to contribute to participants perceptions of contextual fit. Participants described the proposed four week format as straightforward and manageable within military routines, suggesting that reduced time demands may be particularly relevant in contexts characterized by variable schedules and competing occupational responsibilities. Similar considerations have been reported in research with veteran populations, where concise and remotely delivered mindfulness interventions have been identified as potentially facilitating participation (Shue et al., 2023). From a counseling perspective, these findings highlight the importance of adapting intervention delivery to occupational rhythms and organizational constraints rather than assuming that standard intervention formats can be transferred directly to military settings.

Confidentiality and stigma emerged as closely interconnected considerations. Participants consistently emphasized the importance of privacy, while also describing concerns that engagement with psychological support could be interpreted by others as a sign of weakness or reduced professional capability. These concerns are consistent with evidence identifying stigma and fear of judgment as barriers to mental health help seeking in military and veteran populations (Kulesza et al., 2015; Sharp et al., 2015). In this context, confidentiality appears not simply as a logistical requirement but as an important condition shaping whether personnel may feel comfortable considering participation in a psychologically oriented program.

At the same time, participants described institutional and leadership support as important for legitimizing the proposed intervention. This introduces a relevant tension between organizational endorsement and individual privacy. On the one hand, visible support from leadership was perceived as potentially increasing the legitimacy of the program within the military organization. On the other hand, participants also emphasized the importance of discretion, confidentiality, and voluntary participation. These findings suggest that future implementation may require a balance between institutional endorsement and protection of individual autonomy. Previous military research similarly indicates that leadership behavior is associated with help seeking attitudes and that supportive leadership may reduce self-stigma and facilitate willingness to access mental health services (McGuffin et al., 2021). However, the present study did not evaluate whether leadership endorsement would influence actual uptake.

Participants also tended to frame the proposed MBCT program in terms of performance, self-regulation, focus, and resilience rather than treatment of psychological pathology. This performance-oriented interpretation may reflect the perceived alignment of the intervention with established military values and occupational priorities. Previous research has similarly suggested that positioning psychological interventions as resources for performance and resilience may increase their perceived compatibility with military culture (Hepner et al., 2022). In the present study, however, this framing should be understood as a participant preference rather than evidence that performance based presentation would necessarily increase participation. It is also important to note that stress and burnout were explicitly included in the way the proposed intervention was presented to participants. Consequently, favorable expectations regarding potential effects on burnout should be interpreted cautiously, as they may partly reflect the framing of the interview rather than independently generated judgments about intervention effectiveness.

Delivery format was another important consideration. Participants viewed online access as potentially useful for reducing logistical barriers associated with travel and fixed locations, particularly in the context of irregular duty schedules. At the same time, some participants expressed a preference for face to face delivery, indicating that digital delivery was not universally preferred. This variation suggests that format preferences may depend on individual circumstances and that flexibility in delivery could be more relevant than reliance on a single modality. Evidence from veteran populations similarly indicates that accessibility and usability can influence engagement with mindfulness-based programs (Chen et al., 2023).

Scheduling constraints were closely related to these delivery preferences. Participants described unpredictable work rhythms, duties, and operational requirements as potential barriers to participation. Previous research has likewise identified scheduling difficulties and inflexibility as relevant barriers in military and veteran health interventions (Madril, 2021; Fikretoglu et al., 2022). Accordingly, participants accounts suggest that options such as rescheduling or alternative access arrangements may warrant consideration during future intervention development, although their effects on actual engagement remain to be evaluated.

Facilitator credibility also emerged as an important aspect of perceived contextual fit. Many participants preferred a military psychologist who understood military routines, organizational norms, and occupational pressures, while others considered an external facilitator acceptable if confidentiality could be assured. This variation suggests another tension between cultural familiarity and perceived independence. Previous research supports the relevance of cultural congruence and familiarity with military norms when delivering psychological interventions in military populations (Coleman et al., 2017; Clary et al., 2023). Jha et al. (2025) similarly emphasized the importance of instructors understanding military contexts when implementing mindfulness-based training.

Taken together, the findings suggest that prospective acceptability is shaped not by a single program characteristic but by the interaction between intervention design and organizational context. Brevity, flexibility, confidentiality, institutional legitimacy, voluntary participation, delivery format, and facilitator credibility were perceived as interconnected rather than independent conditions. Importantly, several of these considerations involved competing priorities, particularly institutional endorsement versus privacy, organizational legitimacy versus voluntariness, and cultural familiarity versus perceived independence. These tensions provide relevant directions for future development and should be examined empirically in future studies involving direct implementation of the proposed program.

### 4.1. Significance

This study provides qualitative evidence regarding the prospective acceptability of a proposed brief online MBCT program among active-duty military personnel and identifies perceived conditions relevant to its potential future implementation. Participants generally viewed the proposed program favorably, while highlighting flexibility, confidentiality, leadership support, and cultural fit as important considerations. These findings should be understood as prospective perceptions and do not establish actual feasibility, adherence, retention, or intervention effectiveness.

### 4.2. Limitations and Future Studies

Several limitations must be acknowledged. Although the sample included participants from different ranks and service years, all were members of the Portuguese Army and recruited from a single geographic region, which may not reflect the perspectives of personnel from other branches or operational contexts. In addition, the predominance of male participants reflects the gender composition of the Army but may have constrained the representation of women’s experiences. Although reflexive procedures were incorporated throughout data collection and analysis, the possibility of social desirability bias cannot be excluded given the military affiliation of the principal investigator.

An important limitation is that participants evaluated a description of the proposed intervention rather than experiencing the intervention itself. Consequently, the findings reflect prospective acceptability and perceived implementation considerations and cannot establish actual acceptability, feasibility, adherence, retention, or intervention effectiveness. These outcomes should be examined in future studies involving direct implementation of the program.

Furthermore, participation was voluntary, and it is possible that individuals more open to psychological interventions were overrepresented, potentially contributing to more favorable perceptions of the proposed MBCT program. Although the analysis was conducted in Portuguese, the translation of selected participant quotations into English may have resulted in minor loss of linguistic nuance despite review against the original excerpts.

Finally, as a qualitative study focused on prospectiveacceptability and perceived implementation considerations, the findings cannot be generalized or used to infer intervention efficacy.

Future research should include larger and more diverse samples and evaluate actual acceptability, feasibility, engagement, adherence, retention, and psychological outcomes during direct implementation of the program. Future studies should also examine how leadership endorsement and institutional context influence participation.

## 5. Conclusions

This study foregrounded the perspectives of active-duty military personnel by exploring their views, perceived barriers, and facilitators regarding a proposed brief online MBCT program prior to implementation. Involving participants at this early stage can provide relevant information for the development and contextual adaptation of psychological interventions in health, organizational, and counseling settings.

Participants generally expressed favorable views toward the proposed brief online MBCT program and identified confidentiality, flexibility, institutional support, workload, stigma, and facilitator credibility as important considerations for its potential implementation within the Portuguese Army. As the intervention was not delivered, these findings represent prospective acceptability and perceived implementation considerations rather than evidence of actual feasibility or effectiveness. Future research should therefore evaluate these outcomes through direct implementation of the program, including actual acceptability, feasibility, engagement, adherence, retention, and potential psychological outcomes.

## Figures and Tables

**Table 1:** Demographic and organizational characteristics of the sample.

Characteristics	*M* (SD)
Age	40 (12.9)
Service Years	18.40 (12.8)
**Characteristics**	**n (%)**
Sex	
Male	11 (73.3)
Female	4 (26.7)
Marital Status	
Single	5 (33.3)
Married	7 (46.7)
Divorced	3 (20)
Education	
High School	9 (60)
College Degree	6 (40)
Service Model	
Contract	6 (40)
Permanent	9 (60)
Military Rank	
Enlisted	5 (33.3)
NCO	5 (33.3)
Officers	5 (33.3)

**Table 2:** Main themes of the thematic analysis.

Themes
Experience and Contact with Psychological Services
Contact with psychologists during service
Training in psychology or military courses related to the subject area
No contact or background
Prospective Acceptability of the Proposed Online MBCT
Prospective mental health benefits
Dynamic, objective, and structured
Privacy and confidentiality
Simplicity, noticeable, and accessible
Practical and Cultural Barriers
Stigma and prejudice
Lack of support and pressure from superiors
Lack of privacy or conditions
Lack of time/always available for duty
Conditions for Implementation
Support from superiors
Dynamization (Individual vs. Group)
Schedule and institutional implementation
Legitimacy (scientific basis, trainers, and technical authority)
Participation (Voluntary vs. Mandatory)
Format and Facilitation Preferences
Psychologist (Military vs. Civilian)
Participation method (Online vs. In-person)
Implementation location (in person)

## Data Availability

The data that supports the findings of this study are available from the corresponding author upon reasonable request.

## Supplementary Materials

The following supporting information can be downloaded at: https://www.mdpi.com/article/10.3390/bs16101821/s1, Supplemental Item S1: JARS–Qual: Information Recommended for Inclusion in Manuscripts That Report Primary Qualitative Research; Supplemental Item S2: Interview Script; Supplemental Item S3: Description of the Sessions of the Brief Online MBCT Program.
