# Supplementary material for: Prospective Acceptability and Implementation Considerations of a Proposed Brief Online Mindfulness-Based Cognitive Therapy Intervention Among Military Personnel
Source: Behav Sci (Basel). 2026 Oct 5;16(10):1821. doi: 10.3390/bs16101821 (PMC13701892; doi:10.3390/bs16101821)
Supplement: Supplementary file 1 [file behavsci-16-01821-s001.zip › Supplemental Item S2.pdf]

Interview script

1. **Section: Introduction (<5 minutes) – No Questions**

   1.1: Expression of gratitude and brief explanation of the purpose: to explore perceptions of the acceptability and applicability of a brief online MBCT program for military personnel with burnout.

   1.2: Ensure confidentiality and, if necessary, reiterate the rights stated in the informed consent.

2. **Section: Experience and attitudes toward psychological interventions (5–7 minutes) – 2 Questions**

   2.1: Do you have any experience with psychological support sessions or wellbeing programs? How was it?

   2.2: What made you feel comfortable or uncomfortable about engaging in online mindfulness-based interventions?

3. **Section: Presentation of the program (5 minutes) – 2 Questions**

   Read: The program consisted of one synchronous online session per week, each lasting approximately one hour, delivered over a four-week period (a total of four sessions) and facilitated by a trained therapist with appropriate adaptations to the original protocol. Each session addressed a specific mindfulness theme and included two to three practical exercises, followed by a guided reflection phase conducted according to the MBCT inquiry process. Sessions also incorporated a review component, during which participants discussed their experiences with mindfulness practice and the application of related skills in their daily routines over the preceding week. Each session concluded with an emphasis on therapist-assigned home practices in the form of brief exercises, designed not to exceed a total of 30 minutes per week.

   3.1: What are your first impressions?

   3.2: What do you find most appealing, and why?

4. **Section: Acceptability and applicability (7–8 minutes) – 5 Questions**

   4.1: How would you feel about participating: comfortable, curious, apprehensive?

   4.2: What challenges do you foresee (duties/exercises/shifts)?

4.3: Does the program's structure make sense to you, or does it seem confusing?

4.4: Do you think it could help reduce burnout or stress? Why?

4.5: Do you feel the techniques could be applied in daily life?

5. **Section: Opportunity and ethics (5 minutes) – 1 Question**

   5.1: Is there any factor (time, career, ethical principles) that would prevent you from participating? Why?

6. **Section: Barriers and facilitators to participation (7 minutes) – 2 Questions**

   6.1: What practical obstacles exist (in addition to those mentioned in the last question)?

   6.2: P: What would make it easier for you to participate (leadership support, anonymity/confidentiality, flexible scheduling)?

7. **Section: Adaptation suggestions (5 minutes) – 3 Questions**

   7.1: What adaptations or examples should be included to make the program more suitable for the Army?

   7.2: Do you prefer individual or group format? Online or in-person modality? What would be the ideal duration or number of sessions?

   7.3: Who should facilitate the program (clinical psychologist external to the institution, military psychologist with appropriate training, or clinical psychologist internal to the institution)?

8. **Section: Implementation (5 minutes) – 2 Questions**

   8.1: Where do you think it would be most feasible to offer the program (Operations/Tecnical Units, Health Units, or an external location)?

   8.2: What organizational steps would be necessary for successful implementation?

9. **Section: Closing and additional feedback (2 minutes) – No questions**

   9.1: Expression of gratitude, opportunity to add any final comments, reiteration of confidentiality.

<u>Total estimated duration (maximum): 45 minutes.</u>
