# Supplementary material for: Prospective Acceptability and Implementation Considerations of a Proposed Brief Online Mindfulness-Based Cognitive Therapy Intervention Among Military Personnel
Source: Behav Sci (Basel). 2026 Oct 5;16(10):1821. doi: 10.3390/bs16101821 (PMC13701892; doi:10.3390/bs16101821)
Supplement: Supplementary file 1 [file behavsci-16-01821-s001.zip › Supplemental Item S3.pdf]

Supplemental Item 3

Figure 1

Description of the sessions of the brief online MBCT program

Session	Themes	Content	Adaptations	Suggested homework
1	Awareness, automatic pilot, and living in our head	• Raisin exercise<br>• Psychoeducation of CBT theory (walking down the street exercise)<br>• Body scan (15 min)	• Mindful breathing was described but not practiced	• Body scan (15 min)<br>• Mindful breathing (10 min)<br>• Pleasant events calendar<br>• Mindfulness of routine activity<br>• Mindful eating
2	Gathering the scattered mind, recognizing aversion, and staying present	• Sitting meditation (breath, body, sounds, thoughts) (15 min)<br>• Automatic thoughts<br>• 3-min breathing space	• Mindful walking and stretching exercises were described but not practiced	• Alternate mindful sitting and stretching or walking (15–20 min)<br>• 3-min breathing space regular and responsive<br>• Unpleasant events calendar
3	Allowing/letting be, thoughts are not facts	• Sitting with difficulties meditation (breath, body, sounds, thoughts, difficult thought or sensation) (15 min)<br>• 3-min breathing space<br>• Turning toward the difficult<br>• Moods, thoughts, and alternative viewpoints exercise<br>• Early warning signs, coping plan	• Breath, body, sounds, and thoughts shortened to a couple of minutes each, followed by sitting with a difficulty, ending with 3-min breathing space	• Sitting meditation or sitting with difficulty meditation (15 min)<br>• 3-min breathing space regular and responsive<br>• Finish early warning signs/coping plan handout
4	How can I best take care of myself?	• Sitting with difficulties meditation (breath, body, sounds, thoughts, difficult thought or sensation) (15 min)<br>• Nourishing/draining exercise<br>• 3-min breathing space-action step	• Body scan and final exercise (stone or loving-kindness) not done	• Plan for daily mindfulness

Note. Retrieved from Luberto, C. M., Wasson, R. S., Kraemer, K. M., Sears, R. W., Hueber, C., & Cotton, S. (2017). *Feasibility, acceptability, and preliminary effectiveness of a 4-week mindfulness-based cognitive therapy protocol for hospital employees. Mindfulness, 8*(6), 1522–1531. https://doi.org/10.1007/s12671-017-0718-x
